# Design of a Modularized IoT Multi-Functional Sensing System and Data Pipeline for Digital Twin-Oriented Real-Time Aircraft Structural Health Monitoring

**DOI:** 10.3390/s25216531

**Published:** 2025-10-23

**Authors:** Shengkai Guo, Andrew West, Jan Papuga, Stephanos Theodossiades, Jingjing Jiang

**Affiliations:** 1 Department of Aeronautical and Automotive Engineering, Loughborough University, Loughborough LE11 3TU, UK; s.guo2@lboro.ac.uk; 2 Wolfson School of Mechanical, Electrical and Manufacturing Engineering, Loughborough University, Loughborough LE11 3TU, UK; a.a.west@lboro.ac.uk (A.W.); s.theodossiades@lboro.ac.uk (S.T.); 3 EVEKTOR spol. s r. o., Letecká 1008, 686 04 Kunovice, Czech Republic; jpapuga@evektor.cz

**Keywords:** structural health monitoring, IoT sensing node, digital twin, real-time

## Abstract

A modular, multi-functional (encompassing data acquisition, management, preprocessing, and transmission) sensing (MMFS) system based upon the Internet of Things (IoT) paradigm is discussed in this paper with the goal of continuous real-time, multi-sensor and multi-location monitoring of aircraft (including drones) structural performances during flight. According to industrial and system requirements, a microcontroller and four sensors (strain, acceleration, vibration, and temperature) were selected and integrated into the system. To enable the determination of potential in-flight failures and estimates of the remaining useful service life of the aircraft, resistance strain gauge networks, piezoelectric sensors for capturing structural vibrations and impact, accelerometers, and thermistors have been integrated into the MMFS system. Real flight tests with Evektor’s Cobra VUT100i and SportStar RTC aircraft have been undertaken to demonstrate the features of recorded data and provide requirements for the MMFS functional design. Real flight test data were analysed, indicating that a sampling rate of 1000 Hz is necessary to balance representation of relevant features within the data and potential loss of quality in fatigue life estimation. The design and evaluation of the performance of a prototype (evaluated via representative stress/strain experiments using an Instron Hydraulic 250 kN machine within laboratories) are detailed in this paper.

## 1. Introduction

Structural Health Monitoring (SHM) [1] is the term used for continuously assessing the condition of structures such as bridges [2,3], buildings [4] and ground transportation vehicles [5] and aircraft [6,7,8]. Its primary purpose is to detect and assess damage, deterioration and other structural anomalies that could compromise the integrity, safety and performance of the structure. By monitoring key parameters, such as vibrations, strains and temperatures, SHM systems can provide early warnings of potential issues, allowing for timely maintenance or repair actions to be taken. This approach has the potential to reduce the cost and time of manual inspection and could also provide prompts to users in case of significant damage in the early stages, thereby reducing the risk of failure. Companies can schedule repairs or replace components based on the sensor data and inferred damage potential, thus extending the service life of the structure at a reduced cost.

**Figure 1:**
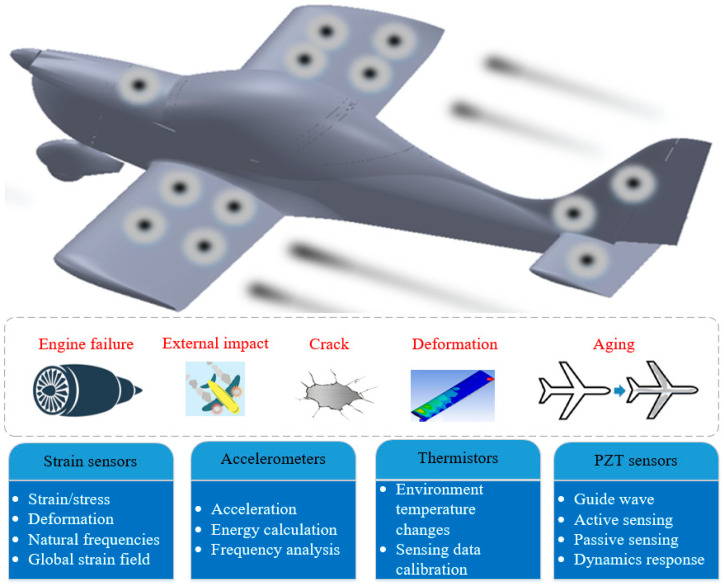
Background of aircraft Structure Health Monitoring (SHM).

With the widespread application of drones [9] for climate monitoring and national defence, the level of intelligence within the aircraft [10] is continuously being enhanced. Additionally, research into the performance monitoring of aircraft during flights is required to enable safety and maintenance to be enhanced. Among the research application scenarios of structural health monitoring, aircraft health monitoring has been reported in the academic literature in recent years [11,12]. Evidence suggests that monitoring various locations of an aircraft can provide valuable information for assessing the aircraft’s health condition [13,14]. Aircraft may encounter various failure scenarios or complex environmental changes during flight, such as wing impacts, cracks, abnormal engine speed and failure, deformation and ageing (see Figure 1). By employing sensors to measure real-time information such as the strain (and hence stress), vibration, deformation, temperature, speed, and acceleration of aircraft structures, structural issues with the airframe could be identified prior to failure. Major aircraft manufacturers like Boeing and Airbus have proposed full-scale fatigue tests and diverse SHM technology [15,16] in aircraft health monitoring, emphasising requirements such as large-scale deployments, lightweight sensors and processing equipment, low power consumption and continuous online monitoring.

Engine failures, external impacts from objects (such as birds and meteorites), cracking at different locations of aircraft, deformation, and ageing are common issues that can affect aircraft structures [17]. Strain gauges play a crucial role in detecting strains, determining stress, and thus also in quantifying load spectra, describing the common service conditions [18]. Accurate strain gauges with consistent response are readily available (e.g., National Instruments, Vishay Precision Group) and thus are commonly used in SHM [19]. Furthermore, Lead zirconate titanate (PZT) sensors can be used in both active (i.e., generating and receiving guided waves to detect structural defects) and passive (i.e., detecting acoustic emissions or vibration signals from crack propagation or impact events without external excitation) sensing modes for aircraft health monitoring [20,21]. In passive sensing mode, PZT sensors are capable of detecting changes in pressure, strain, force and flight dynamics. In active sensing mode, PZT sensors are split into two parts: PZT actuators, which act as transmitters and PZT sensors, which act as receivers. Accelerometers can be utilised to monitor and classify abnormal vibrations at specific aircraft locations, detecting potential faults in aircraft engines and hence supporting the reduction in in-flight issues. In addition, research has shown that the combination of accelerometers and strain gauges can improve the accuracy of dynamic displacement measurements [22].

Temperature has to be monitored to determine its impact on performance at different altitudes [23]. Moreover, strain signatures detected using strain gauges could vary under different temperature conditions depending on the temperature coefficient of resistance variations and strain gauge deployment configurations [24]. Therefore, temperature sensors are necessary to enable compensation for temperature drift and ensure accurate strain measurements.

The concept of a digital twin (DT), originally proposed by Air Force Research Laboratory over a decade ago [25], has been widely used in various industrial sectors [26,27], including civil SHM [28,29,30,31], aircraft SHM, and other related domains [32]. At its core, DT involves creating a virtual replica or “twin” of a physical asset, leveraging data from physical models, sensor updates and operational histories [33]. This virtual representation can be used to integrate multidisciplinary, multiscale and probabilistic simulation processes to map the entire lifecycle of the corresponding physical equipment. Digital twinning exhibits three key characteristics: full lifecycle coverage, real-time interoperation and bidirectional communications [27,33]. In the context of aircraft SHM, a DT offers a promising approach as it can incorporate various data sources to calculate the health status and remaining useful life (RUL) of the aircraft and its components [34] using data-driven pattern recognition methods (e.g., machine learning and deep learning). Unlike SHM approaches in other scenarios, real-time acquisition of sensor data is crucial during aircraft flight, necessitating the operation of any DT functionality in real-time.

**Figure 2:**
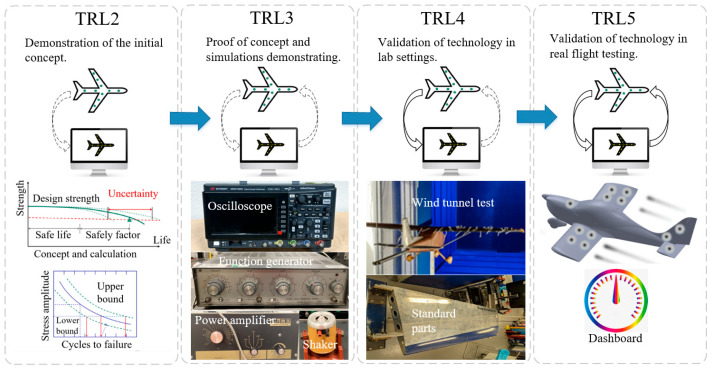
TRL of digital twin platform of aircraft SHM.

The technology readiness level (TRL) of the aircraft SHM digital twin platform can be viewed as progressing through a number of stages, from concept validation (i.e., TRL 2) to real flight conditions (i.e., TRL 5) as depicted in Figure 2. At TRL 2, the concept and validation methods are established [35], while TRL 3 involves proof of concept in simple experimentation [36] and via simulations. Advancing to TRL 4 and 5 requires increasingly realistic experimental conditions, including experiments in laboratory wind tunnel environments and real flight conditions, respectively, both involving real-time data acquisition [37].

Some of the literature on structural health monitoring has proposed distributed sensing subsystems for monitoring multiple parts of a structure [38,39]. However, these designs are primarily intended for civil infrastructure, environmental monitoring, and healthcare applications, rather than aircraft sub-systems. As a result, key aspects such as sub-system architecture, core modules, deployment models, and implementation methods remain insufficiently detailed for aerospace contexts [40]. Furthermore, some of the reported sensor nodes are not suitable for direct extension to multiple parts of an aircraft. Most importantly, reported designs often lack validation using real flight test data; hence, multiple factors such as the sampling rate of sensors, the sensing measurement range at different flight stages and external impacts and damage are not addressed during the sensing node design stage. This is also one of the reasons why real flight test data has to be the focus of practical SHM for aircraft.

To obtain real-time sensor data from multiple components of an aircraft’s structure, a multi-sensor subsystem monitoring strain, acceleration, dynamic load and temperature is proposed in this research. Each subsystem is designed in a modular fashion, facilitating scalability to monitor a larger area and more components of the aircraft [41]. Additionally, the hardware and software components are designed to support continuous and real-time sensor data retrieval. To meet the requirements of both the aircraft and digital twin systems, the data acquisition frequency of the multi-sensor subsystem has been analysed based on acceleration data collected from several real-world flight tests. The results are applied to the extended multi-sensor subsystem, resulting in the design of a modular Internet of Things (IoT) multi-functional sensor system tailored for real-time health monitoring of aircraft during flight in the digital twin environment.

Compared with conventional SHM/IoT-based monitoring systems, the proposed hardware layer introduces a modularised and reconfigurable design specifically tailored for digital-twin-oriented applications. It enables synchronised multi-sensor acquisition (strain, vibration, acceleration and temperature) with on-board pre-processing and real-time transmission, which are essential for model-driven digital twin updates.

The main contributions of this paper are as follows:

(1) A modularised, non-customised design approach for multifunctional sensing systems, which can be adapted and applied to various forms of machines (including manned aircraft and drones).
(2) A sensing system that can be utilised to monitor strain/stress values, dynamic characteristics, acceleration, acoustic emission and temperature simultaneously and continuously throughout complete flights.
(3) Whilst other research has been focused on design, development and analysis of theoretical concepts, the research reported in this paper is focused on practical applications targeting real-time deployments on small aircraft and drones.

## 2. Design and Operating Principles

**Figure 3:**
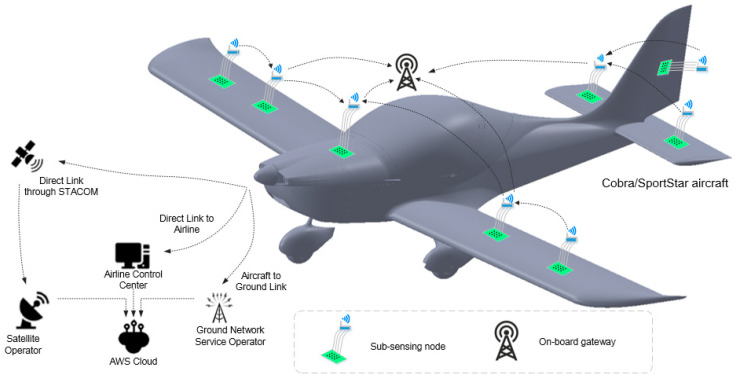
Overall data connectivity architecture for data collection from aircraft to Cloud.

The data connectivity architecture for the aircraft health monitoring system is shown in Figure 3. The physical system consists of a series of sub-sensing nodes, an on-board gateway and aircraft components, while the virtual system is represented by a digital system in the Amazon Simple Storage Service (Amazon S3), a part of the AWS Cloud [42,43]. Data transfer occurs between these physical systems and virtual systems, with the goal of secure communication for inspection control, data handling and storage. There are two main types of connections in the proposed data communication platform (as shown in Figure 3):

- **Intra-Air Vehicle Connection:** This connection links the IoT sensors with the on-board aircraft gateway;
- **Aircraft-to-Ground Connection:** This connection is between the aircraft and ground and is defined to support interconnection between the physical aircraft system with the virtual digital twin potentially hosted via cloud services [43].

Distributed sensor nodes are to be deployed across multiple critical areas. Multiple nodes can be placed on the main wings to assess the structural health of key locations during flights. Scalability is key to adapting the number of sensor nodes in all locations as requirements change. Sensor nodes located on the main wing, engine, engine nacelle, tail wing and centre of gravity are integrated into a unified sensor network. The on-board gateway is designed to manage all distributed sensing nodes, supporting synchronisation of sensor data. Non-customised sensing nodes (not only commercially available off-the-shelf sensors, but, e.g., printed strain gauges) can also be wirelessly integrated into the network. All data collected from these nodes are transmitted to a cloud-based platform via satellite network, where it is processed, analysed and incorporated into the digital SHM twin. If required, key results can be fed back to the pilot for assessment and actioning.

### 2.1. System Architecture and Function

**Figure 4:**
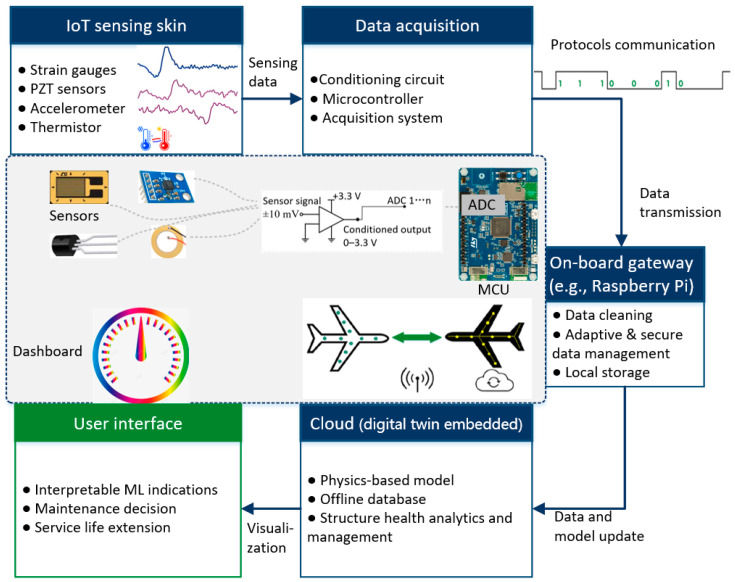
Digital twin-enabled SHM platform for aircraft: system interconnection schematic.

Figure 4 presents how the sensor data from various sensing nodes are acquired, processed, computed, stored and utilised by the digital twin platform to fulfil the functionalities required for the physical system illustrated in Figure 3. As the current STM32L4 series Microcontroller Unit (MCU) can only measure voltage input signals in the range of 0–3.3 V, any signals outside of this range should be scaled through conditioning circuits to ensure compatibility with the MCU’s input limits [44]. The data acquisition system processes the collected sensor data (i.e., strain, acceleration, temperature, and vibration) and transmits it to the onboard gateway via a number of selectable communication protocols (e.g., Wi-Fi, Zigbee, and BLE protocols [45]). Subsequently, the goal is that all of the data are transmitted to and managed within the cloud [46], where the digital twin will be constructed and embedded. The digital twin platform retrieves the latest sensor data from the cloud, updates the database, computes the health status and finally provides feedback to users regarding, for example, the structural health parameters and remaining useful life estimate.

### 2.2. Multi-Sensing Subsystem Definition and Specification

**Figure 5:**
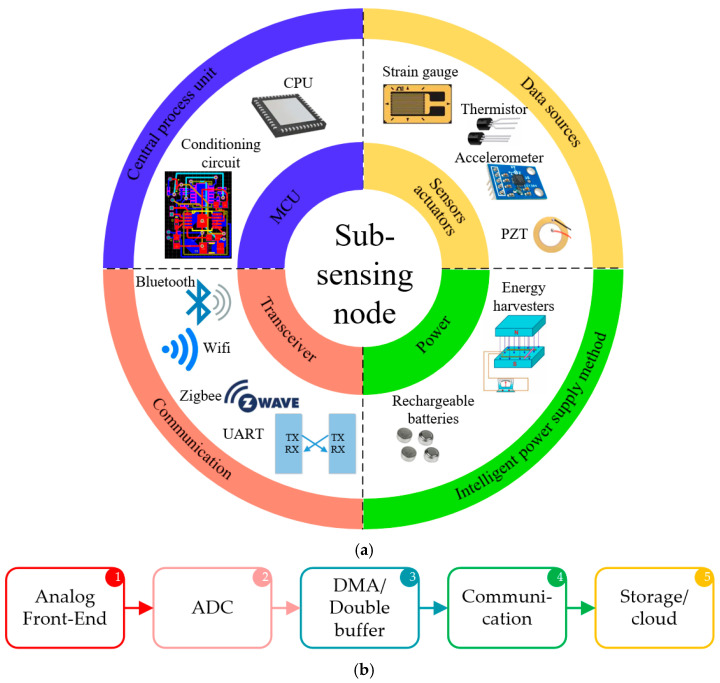
(**a**) Sub-sensing node system components and (**b**) system-level link budget diagram.

According to the requirements of the aircraft SHM digital twin platform, sub-sensing nodes consist primarily of four components: an MCU, one or more sensors and actuators, a transceiver and a power supply (e.g., rechargeable batteries and vibration energy harvesters), as is shown in Figure 5a. In this study, the B-L475E-IOT01A discovery board was chosen as the central processing unit [47]. The selected MCU is STM32L475VG, which is characterised by a maximum processing clock speed of 80 MHz, 16 Analogue-to-Digital Converter (ADC) channels and two DAC channels. It also includes 1 Mbyte of Flash memory and 128 Kbytes of SRAM. Additionally, the discovery board can supply 3.3 V to the sensors.

The EVAL-ADXL 356CZ 3D accelerometer provides a measurement range from −40 g to 40 g in three axes [48]. The operating voltage of this accelerometer is 3.3 V, which enables it to be powered directly by the discovery board.

The uniaxial strain gauge module incorporates the BF350-3AA strain gauge [49], featuring an integrated amplifier and potentiometer to adjust the measured results for offsets, drift and deployment conditions (e.g., cable lengths). The measurement range of the selected strain gauge is ±2% [50]. This indicates that the strain gauge can measure strain within a range of ±2% of the material’s original length, equivalent to ±20,000 micro strain value (με). The operating voltage of this module is 3.3 V, allowing it to be powered directly by the discovery board. Piezoelectric (PZT) sensors with “4.6 kHz Standard Buzzer Element 1.063” Dia (27.00 mm) and 300 Ohms resistance are utilised for monitoring aircraft dynamics. Because alternating voltage is generated when an impact is detected, a conditioning circuit is needed to adjust the sensor’s raw output voltage (e.g., from −100 V to +100 V) to a level (i.e., 0–3.3 V) that can be directly read by the MCU’s ADC channels.

The selected integrated temperature sensor, LM35, is an analogue sensor with a temperature range of 0 °C to 150 °C and a linear scale factor of +10 mV/°C [51]. It requires a 3.3 V power supply; hence, it can be powered directly by the discovery board.

The MCU supports a number of communication modes and modules [47] on board, including Bluetooth Low Energy (BLE), WIFI and Universal Asynchronous Receiver/Transmitter (UART), providing flexibility in communication methods. For example, when transmitting data via UART, the data are directed to the transmit (TX) components of the UART serial port and then sent to the on-board gateway. In addition to transmitting sensor data, preprocessing functionalities such as noise reduction and smoothing (e.g., via digital filters [52]) need to be embedded within the MCU.

Power management software functionality and rechargeable batteries are integrated to support the operation of the sub-sensing system. Furthermore, a stable and reliable power supply method is also essential to ensure the continuous functionality of the sensing subsystem. Rechargeable batteries can provide stable power to sensors, MCUs, communication modules, and other system components (e.g., conditioning circuit). Within this system, energy harvesters (e.g., piezoelectric or electromagnetic energy harvesters) are strategically positioned in specific areas of the aircraft, such as the wings and engine mounts, where significant vibrations are observed during flight. These harvesters employ piezoelectric materials and electromagnetic induction to convert vibrational energy into electrical energy [53]. The harvested energy can then be stored (via capacitors, e.g., 330 μF supercapacitor [54]) or used to power sensing nodes. This capability may allow low-power/low operational cycle electronic components/sensors to be partially self-powered. The Power Management Module [55] manages the charging and discharging strategies for rechargeable batteries and energy harvesters. By implementing efficient power management techniques (e.g., Programmable Maximum Power Point Tracking [56]), energy utilisation and operational duration/duty cycles of the sensing subsystems can be optimised.

The proposed MMFS system integrates analogue signal conditioning, digital sampling, and wireless/cloud communication in a unified architecture. A system-level overview of the signal chain is illustrated in Figure 5b, showing the flow of information from the analogue sensor to the final data storage.

The bandwidth of each module is determined by circuit design parameters and sampling configurations (for example, in this paper’s test, ADC sampling rate = 1094 Hz), as is shown in Table 1, while latency values are derived from a combination of STM32L475 datasheet specifications and empirical timing measurements. The analogue front-end determines the system’s effective bandwidth, while the Direct Memory Access (DMA)-based ADC subsystem ensures continuous sampling without loss. Some sensor channel analogy front-ends (such as strain gauges and accelerometers) incorporate filters. Double-buffered DMA minimises data latency and loss, while BLE and SD card layers use checksum mechanisms to ensure data integrity. The end-to-end latency—from sensor analogy signal to stored or transmitted data—is below 50 ms, which is sufficient for low- and mid-frequency structural monitoring and vibration analysis. Data continuity and integrity are ensured at each stage through hardware synchronisation and software-level validation.

## 3. Function Realisation and Implementation

To achieve real-time acquisition and processing of sensor data from a sub-sensing node, appropriate hardware devices have been selected, including four kinds of sensors and MCU development boards. The operational methodology of the data acquisition software has been designed accordingly.

### 3.1. Hardware and Software Implementation

**Figure 6:**
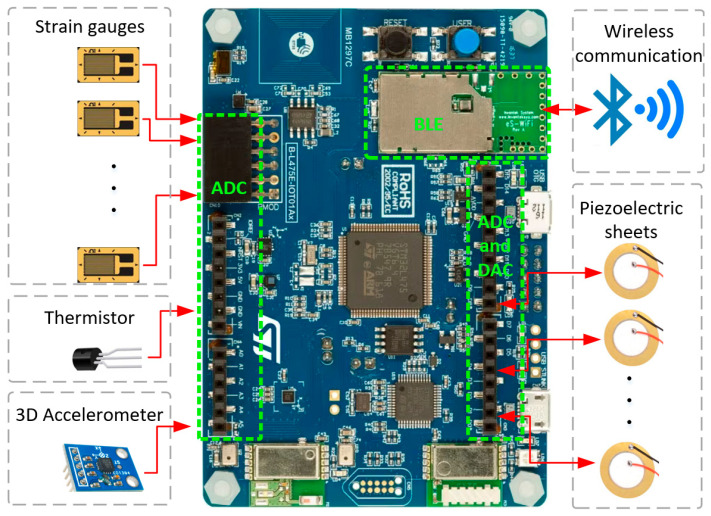
Hardware diagram of a distributed multi-functional sub-sensing node and MCU.

**Figure 7:**
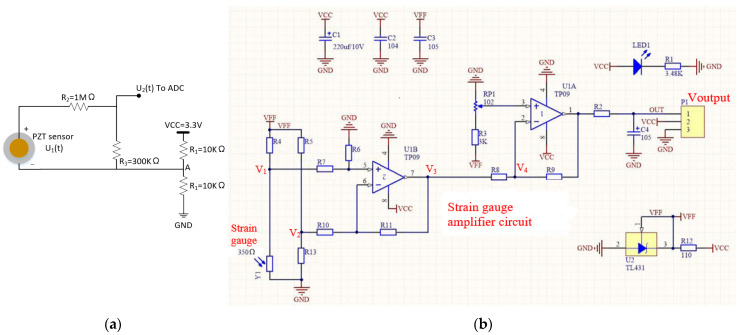
PZT and strain gauge conditioning circuit. (**a**) PZT sensor conditioning circuit. (**b**) Strain gauge amplifier circuit diagram.

Figure 6 illustrates the hardware components of a sub-sensing node. Since all four sensor types used are analogue, data acquisition involves utilising the development board’s built-in ADC channels to read and convert analogue signals into digital values. Figure 7a shows the conditioning circuit for each piezoelectric sensor. By adjusting the resistance values of *R*_1_, *R*_2_, and *R*_3_, the output voltage can be expressed as:

*U*_2_(*t*) = VCC/2 + *U*_1_(*t*)*R_3_/(*R*_2_ + *R*_3_) (1)

This allows the voltage from the piezoelectric element to be properly scaled for input into the ADC channels and subsequently read by the MCU. The ADC channels accept voltages within the range of 0–3.3 V. For example, when employing a 12-bit ADC, the raw values range from 0 to 4095 (i.e., 2^12^ − 1 = 4095). Multiplying the recorded data by 3.3/4095 yields the voltage value detected by the ADC channel. With the established functional relationship between sensor voltage and parameters (i.e., acceleration, strain, vibration, or temperature), sensor data values can be derived. For example, if the ADC channel reads a value of 124 for an LM35 integrated temperature sensor, the current voltage is 100 mV (i.e., 124/4095 × 3.3 V = 0.1 V). By utilising the conversion factor of 10 mV/°C from the manual [51], the current temperature value can be calculated as 100/10 = 10 °C.

(2) $$V_{output}=\frac{R_{8}+R_{9}}{R_{8}}\cdot V_{4}-\frac{R_{6}R_{9}}{R_{7}R_{8}}\left(\frac{Y_{1}}{348+Y_{1}}-\frac{1}{2}\right)\cdot V_{FF}$$

The strain gauge amplifier circuit used in this article is shown in Figure 7b, and its corresponding electrical parameters are listed in Table 2. Based on the electrical parameters in the figure, we can derive the functional relationship between the amplifier circuit’s output voltage signal *V*_output_ and the input strain gauge resistance *Y*_1_. When the strain gauge is not subjected to any strain, its resistance is *Y*_1_0_ (the factory default value). When subjected to a certain strain, the strain is *Y*_1_(*t*). The corresponding resistance change is

(3) $$∆Y(t)=Y_{1}\left(t\right)-Y_{1\_O}$$

Since there is a linear relationship between strain and resistance change in the elastic working range of commercial strain gauges, the real-time strain value can be obtained based on the strain change and gauge factor parameters. For the employed strain gauge amplification circuit, a low-pass filter was implemented in the analogue front end, with a cutoff frequency of *f_c_* = 1/(2*πR*_2_*C*_4_) ≈ 81 Hz. To mitigate temperature-induced zero-drift and sensitivity variations, a dedicated temperature sensor was sampled simultaneously with the strain channel by the MCU. The measured temperature data from the LM35 integrated temperature sensor were used in post-processing to correct thermal effects through a linear compensation model. Specifically, the temperature-dependent offset *V_offset_*(*T*) was modelled as a linear function:

(4) $$V_{offset}\left(T\right)=aT+b$$

where the coefficients *a* and *b* in the equation can be determined from multiple sets of temperature *T* and the corresponding zero-strain output voltage *V_offset_*(*T*). The real-time compensated strain data were then obtained through a post-processing correction:

(5) $$\varepsilon_{c}=\varepsilon_{m}-\alpha_{T}[T-T_{0}]$$

where $\varepsilon_{m}$ is the uncorrected strain derived and calculated from the strain gauge amplifier circuit output, $\alpha_{T}$ is the experimentally determined temperature drift coefficient, and $T_{0}$ is the reference temperature.

Additionally, other ADC modes such as 16-bit, 10-bit, 8-bit, and 6-bit can be selected. Compared with the 12-bit ADC module, the 16-bit ADC offers higher sampling precision enabled by its hardware oversampling mode. Conversely, the 10-bit, 8-bit, and 6-bit ADCs provide lower sampling precision but shorter conversion times (i.e., 10.5, 8.5, and 6.5 ADC clock cycles per conversion, respectively [57]). In this study, a 12-bit ADC is chosen to strike a balance between sampling precision (i.e., 0.8 mV) and conversion time (i.e., 12.5 ADC clock cycles per conversion).

**Figure 8:**
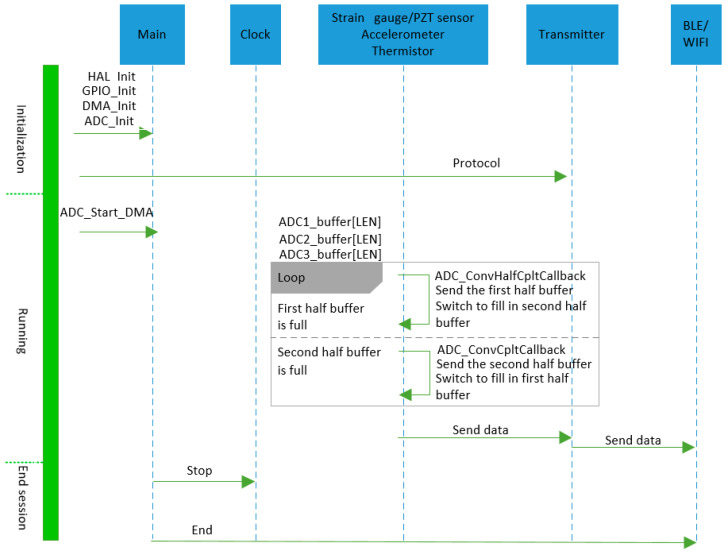
UML sequence diagram of data acquisition software functionality.

A Unified Modelling Language (UML) sequence diagram [58] of the data acquisition software functionality, as depicted in Figure 8, illustrates the chronological sequence of events during the system’s operational cycle. The modules (i.e., clock, sensor signal, transmitter, and BLE/Wi-Fi) listed represent the system participants, while the timeline on the left-hand axis delineates the temporal sequence of programme execution into three phases: initialisation, running, and end session (i.e., termination).

**Initialisation Phase**: In the initialisation phase, the Hardware Abstraction Layer (HAL) library, General Purpose Input/Output (GPIO) ports, bus clock, and communication protocols are set up using library functions. The HAL library, written in C, offers the core functionalities such as activating specific GPIO ports and initialising system states (e.g., configuring clock settings, setting up communication protocols, and managing peripherals). During the ADC initialisation phase, multiple channels are used with continuous conversion enabled. The ADC data is transferred directly to the Direct Memory Access (DMA) channel, which operates in continuous circular mode. Therefore, it is necessary to enable the ADC scan mode and continuous conversion, as well as to enable the DMA channel’s continuous request and circular mode. Communication protocols required for UART, BLE, or Wi-Fi capability are then defined. Users must configure all communication parameters, including baud rate, parity, transmitter and receiver settings (e.g., 115,200 baud rate, no parity, eight data bits, one stop bit, with hardware flow control enabled for a typical UART deployment [59]).

**Runtime Phase**: During the runtime phase, the ADC module retrieves sensor data from defined channels. Data from each ADC channel is sampled using the ADC_Start_DMA function, with each channel’s data stored in a variable with a length of ADC_BUF_LEN (e.g., 1000). Additionally, to support real-time and efficient data processing, interrupt handlers for DMA half-transfer and transfer completion events, namely ADC_ConvHalfCpltCallback and ADC_ConvCpltCallback, are implemented. These callbacks enable timely data export and processing by leveraging a double-buffering mechanism, which allows one half of the DMA buffer to be processed while the other half continues to be populated by ongoing ADC conversions. This approach ensures continuous data acquisition and maintains the real-time integrity of the sampled signals. These functions are re-implemented within the main function to meet operational requirements., During the DMA half-transfer event, the system processes and exports data from the first half of the buffer, while the ADC continues writing to the second half. Conversely, during the DMA transfer completion event, data from the second half is processed, while new samples begin filling the first half again. This cyclical access pattern ensures uninterrupted retrieval of sensor data from ADC channels, which is then transmitted via either UART, BLE or Wi-Fi to local storage and the on-board communication gateway.

**Session Termination Phase**: The final phase is session termination, during which the system ceases data acquisition, stops ongoing DMA transfers, and closes software running on the MCU. Additionally, all peripherals (e.g., ADC, timers, and communication interfaces) are de-initialised, and memory buffers are cleared or released. The system safely shuts down or transitions into a low-power standby mode when required.

### 3.2. Data Acquisition, Management, Preprocessing, and Interpretation

When utilising data from the multi-functional sensing subsystem for aircraft structural health monitoring on the digital twin platform, preprocessing of the data after acquisition by the ADC is necessary. Preprocessing of sensor data includes noise reduction (e.g., high-pass, low-pass filtering algorithms [60]), smoothing [61,62], and wavelet analysis [62].

**Sensing Data Acquisition, Management, and Storage Strategy**: For each MMFS defined in this paper, the sampling of sensor data follows the software part in Section 3.1. The data-volume budget can be estimated as *N_nodes_* * *N_channels_* * *f_s_* * resolution * *T_flight_*. For example, considering two nodes with seven channels each, sampling at 1094 Hz (typical value used in this study) with a 12-bit ADC resolution during a 1.5 h flight (typical value used in this study), the total raw data volume is approximately (two Nodes * seven channels * 1094 Hz * 2 bytes * 1.5 H * 3600 s/H ≈ 165 MB). This volume is well within the on-board SD storage capacity (8 GB, 16 GB, or higher). Meanwhile, key features can be extracted and then transmitted to the ground station, reducing the wireless backhaul demand.

**Signal Analysis and Feature Extraction**: Wavelet analysis helps decompose signals into components of different scales (i.e., low-frequency baseline drift and high-frequency fault components) and frequencies, enabling a better understanding of the signal’s structure (e.g., abrupt impacts and resonance patterns) and characteristics (e.g., damage-related frequency shifts) [63]. Through wavelet analysis, important features such as changes, periodicity, and trends in the signal can be identified.

**Noise Reduction and Signal Enhancement**: Filtering is a common signal processing technique used to remove noise or unwanted components from signals, thereby improving signal quality and clarity. Wavelet filters can locally process signals based on their frequency and scale characteristics, effectively removing noise and potentially enhancing the components of interest.

**Compression and Data Dimensionality Reduction**: Wavelet transforms have the ability to compress signal information by representing signals as smaller sets of coefficients, achieving data dimensionality reduction and compression. For example, the discrete wavelet transform (DWT) has been applied to sensor signals for lossy compression [64], reducing data size while preserving essential features. This is particularly useful for storage, transmission, and processing of large datasets, saving storage space and reducing transmission energy consumption, for instance, by minimising the volume of data that needs to be communicated using wavelet-based coding schemes [65].

**Feature Extraction and Pattern Recognition**: Using wavelet transformations, signals can be transformed into a set of feature parameters with time-frequency characteristics. These parameters can be used for tasks such as pattern recognition, classification and regression [66] to enable fault detection and anomaly recognition [67]. Features extracted through wavelet analysis often contain more information and discrimination power compared with traditional frequency or time-domain analyses [68].

## 4. Preliminary Test Result

### 4.1. Experiment Preparation

**Figure 9:**
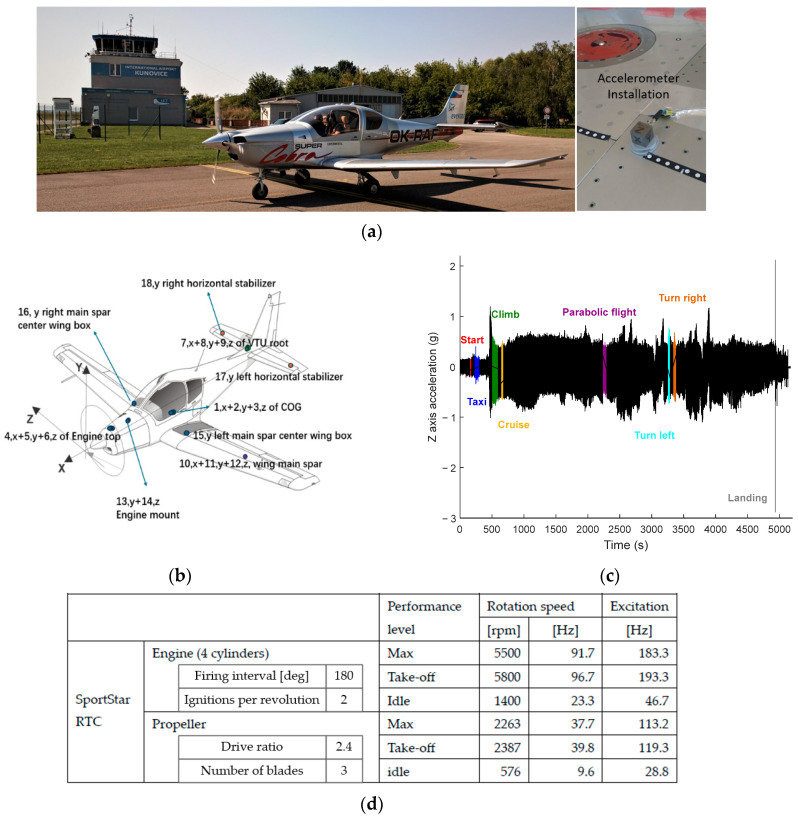
Acceleration data acquisition during real flight tests and the excitation source analysis. (**a**) Real flight test of Evektor‘s Cobra VUT100i aircraft at Kunovice airport. (**b**) Locations and orientations of 18 acceleration measurement channels. (**c**) Time history of acceleration of Engine mount in the Z axis during whole flight (key phases are highlighted by colour). (**d**) Analysis of excitation sources of SportStar RTC that affect the definition of the adequate sampling frequency.

To acquire data during aircraft flights to support the design of the embedded data acquisition system, accelerometers were installed on the Evektor Cobra VUT100i (see Figure 9a, EVEKTOR spol. s r. o., Kunovice, Czech Republic) and SportStar RTC aircraft (EVEKTOR spol. s r. o., Kunovice, Czech Republic) to measure typical acceleration characteristics during different flight phases (e.g., take off, taxi, climb, cruise, and landing). As an example, the Cobra VUT100i aircraft was equipped with a data recording system for 18 accelerometer channels (using, e.g., Kistler 8315A010, Kistler Instrumente AG, Winterthur, Switzerland, or various printed circuit board (PCB) accelerometers), see Figure 9b. The analyses of the excitation sources show that the engine of the smaller SportStar RTC aircraft results in the excitation at nearly 200 Hz (Figure 9d). Sampling frequencies below 1000 Hz result in the loss of high-frequency acceleration features. The signal loss converts to a decrease in the related fatigue life from the computational analysis. Therefore, in the designed multi-sensor subsystem, the sampling frequency of each ADC channel is set to be larger than 1000 Hz to ensure comprehensive data capture.

Each aircraft was flown under various conditions for three flights for an approximate total of 3 h. The schematic diagram of 18 acceleration measurement positions is shown in Figure 9b with a representative example of acceleration data from the engine mount in the Z axis, shown in Figure 9c. Each channel corresponds to a specific location within the aircraft structure and a specific orientation in the aircraft coordinate system.

To observe and understand the response of various segments of the aircraft to differing flight conditions and to see the limits of obtained acceleration values, an experimental sequence of flight states was followed throughout the flight, including start, taxiing, climb, cruise, parabolic flight (i.e., a flight manoeuvre in which the aircraft follows a ballistic trajectory to create short periods of microgravity), turn left, turn right, and landing.

**Figure 10:**
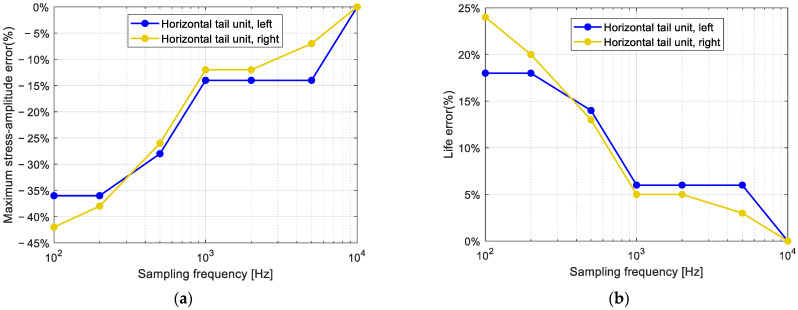
Evaluation of data loss and its projection into the fatigue life analysis for the original recorded signal at 10 kHz sampling frequency. (**a**) maximum stress-amplitude error. (**b**) life error.

To check that the chosen sampling frequency of 1 kHz is a reasonable trade-off between the data volume and loss of precision in the subsequent analysis, another set of in-flight measurements on Cobra VUT100i followed. The acceleration at the tail units was monitored with a sampling frequency of 10 kHz. This signal was used to detect the maximum stress amplitude in the load spectrum and the related predicted fatigue life from the whole strain record. These two parameters were also established for the signal records obtained by decimating the original signal to 100, 200, 500, 1000, 2000, and 5000 Hz. The graph in Figure 10 shows the differences in the maximum stress amplitude in the spectrum and in the calculated lifetime related to the whole record for various sampling frequencies. It provides quite a convincing decision point for accepting 1 kHz as a reasonable compromise between the volume of data and the quality of the obtained fatigue life estimation.

### 4.2. Experiment Setup

**Figure 11:**
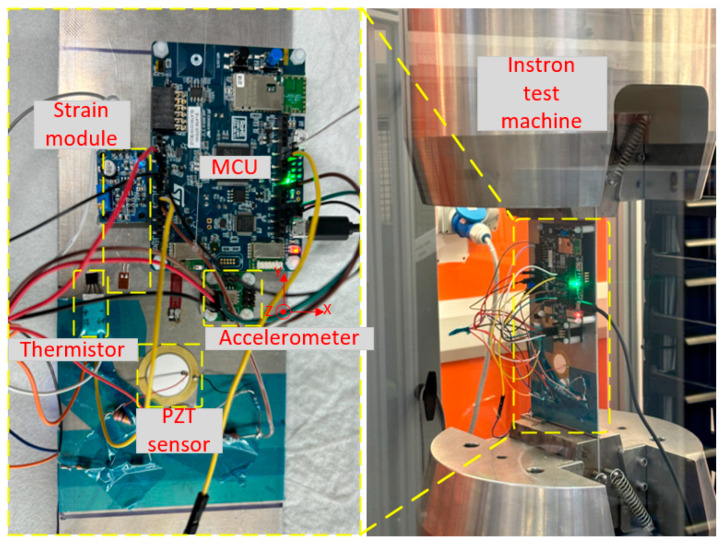
Experiment setup and initial test of the sensing data acquisition system.

The experimental setup for testing the prototype multi-sensing system is depicted in Figure 11. To ensure that the prototype meets the size requirements of the Instron test machine, all components were arranged on a standard aluminium plate (300 mm × 100 mm × 2 mm), including the B-L475E-IOT01A MCU, a strain module containing a uniaxial strain gauge (BF350-3AA) and an amplification circuit, a PZT sensor to measure structural vibrations and impact-induced signals, and a conditioning circuit module that converts the PZT’s AC output into a 0–3.3 V signal suitable for ADC channel sampling, an EVAL-356CZ 3D accelerometer and an LM35-integrated temperature sensor. All modules were attached to the aluminium plate specimen using super glue, with the strain gauge and PZT sensor located near the centre. The ends of the aluminium plate specimen were clamped by the Instron machine. The Instron machine was activated to apply static loads from 0 to 15 KN. A UART terminal software (XCOM or Tera Term) on the laptop was employed to control the interface, receive, and log the sensor data for analysis.

### 4.3. Experiment Results

**Figure 12:**
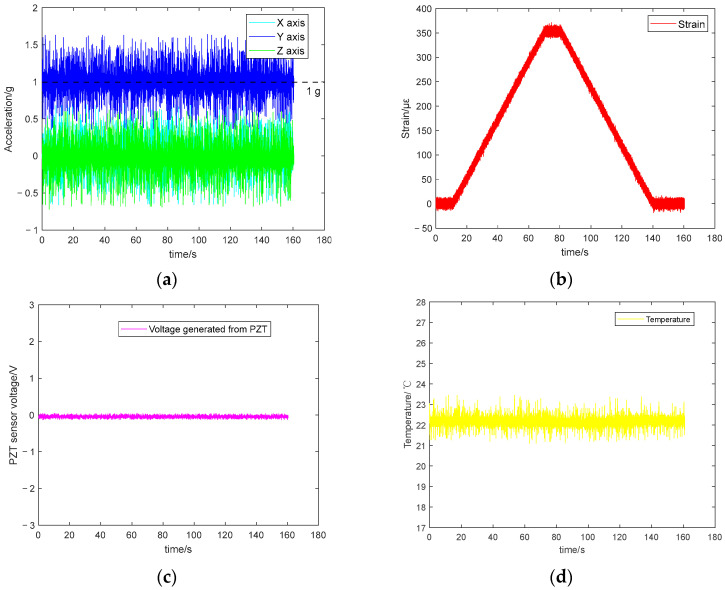
Static loading test results using the Instron machine. (**a**) 3 axes acceleration. (**b**) strain. (**c**) PZT sensing data. (**d**) temperature.

The data for 3D acceleration, strain, PZT voltage, and temperature are presented in Figure 12a–d, respectively. Note: The *Y*-axis of the accelerometer is parallel to the direction of gravity, resulting in a *Y*-axis measurement of 1 g. The sampling frequency of all ADC channels is 1094 Hz, and the ADC resolution is 12 bits.

**Figure 13:**
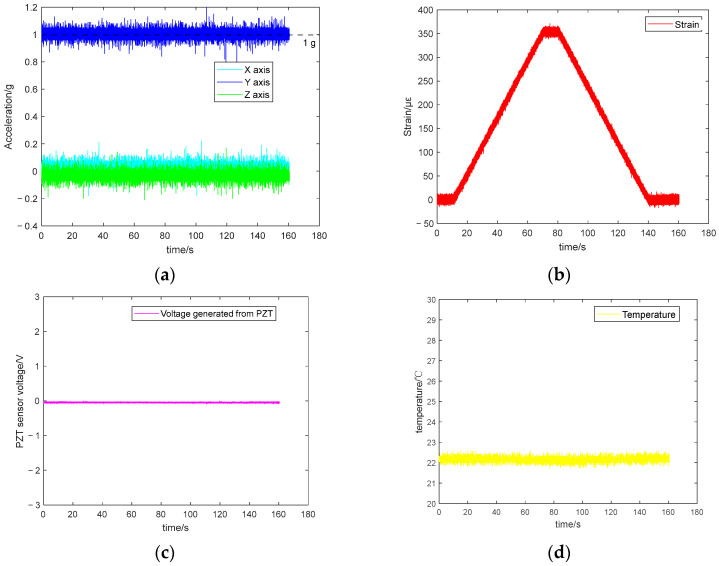
Pre-processed results of the static loading test conducted using the Instron machine. (**a**) 3 axes acceleration. (**b**) strain. (**c**) PZT sensing data. (**d**) temperature.

Notable noise is observed in Figure 12, which may be attributed to factors such as electromagnetic interference. Hampel filtering [69] was applied for denoising and smoothing signals. The filter used a sliding window of three samples and a threshold of three times the median absolute deviation (MAD), where outliers exceeding this threshold were replaced by the local median value. The pre-processed data are displayed in Figure 13. After Hampel filtering, the amplitude of the 3D acceleration noise reduced to 0.1 g, with the *Y*-axis stabilising around 1 g and the X and Z axes stabilising around 0 g. The strain gauge began detecting the static load applied by the Instron machine at around 10 s (see Figure 12b). Between 10 s and 70 s, the strain increased from 0 to 360 με, followed by a static strain of 360 με for 10 s. The static load was gradually reduced to 0 during 80 s to 140 s. Finally, the Instron machine cycle ended at 140 s. Due to the absence of dynamic loads, the output from the PZT remained largely unchanged during 0–160 s. The integrated temperature sensor measurements primarily reflected the ambient room temperature (i.e., 22°).

### 4.4. Discussion and Future Work

(1) PZT Signal Conditioning and Ongoing Improvement. In the current prototype, the PZT output (−100 V to +100 V) was scaled into the MCU’s 0–3.3 V ADC range using a high-value resistor divider for preliminary system integration. However, it inherently loads the high-impedance piezoelectric source and limits the low-frequency response. To address this limitation, a dedicated charge amplifier (transimpedance topology) is currently being designed. The new configuration provides a very high input impedance and a defined frequency response, which helps preserve the original piezoelectric signal and reduce noise. The equivalent circuit and calibration results will be included in future work.
(2) This paper provides a feasible scheme for converting ADC readings into strain data through the modulation/amplification circuit of the strain sensor. However, strain measurements may drift under different temperatures. We propose a temperature compensation strategy based on the real-time temperature data provided by the system itself. In future work, we will further refine this approach through dedicated testing that integrates both temperature and strain data.
(3) In terms of software design, the proposed data acquisition scheme effectively enables the collection of multiple types of sensor data, including strain, acceleration, vibration, and temperature. For structural health monitoring, different sensors have different sampling frequency requirements—for example, piezoelectric and accelerometer channels require relatively high sampling rates, while temperature channels can be sampled at lower rates. Based on our current data acquisition design, optimisation is possible. The MCU used in this work provides multiple ADC modules and channels and supports DMA and timer-triggered modes. By assigning different ADC sampling frequencies to different sensors, the data acquisition can be optimised to better support structural health monitoring and remaining useful life calculations.
(4) In a full digital twin framework, the presented device represents the physical data acquisition layer. It provides the raw and pre-processed data streams required for real-time model updating, state estimation, and life prediction algorithms implemented in the digital domain. Although this paper focuses on the hardware layer, the system is designed to interface seamlessly with higher-level analytics and modelling modules that constitute the complete digital twin. Our next step will be to integrate the proposed MMFS with model-based and data-driven algorithms for fault detection and remaining useful life prediction.

## 5. Conclusions

This design and initial evaluation of a modular, multi-functional sensing system (MMFS) for aircraft structural health monitoring on a digital twin platform are presented in this paper. To meet the real-time and distributed sensing data requirements of digital twin applications, each multi-functional sensor node in the system is connected to a processor and integrates four types of sensors (strain gauges, PZT sensor, accelerometer and thermistor). This modular design facilitates expansion to include more areas of the aircraft fuselage within the SHM system. To ensure that the data from the sensor nodes could sufficiently meet the requirements for aircraft damage assessment without losing critical information, a real flight test was conducted using an Evektor’s Cobra VUT-100i aircraft. The analysis of sensor data collected during the actual flight defined the sampling frequency for the MMFS. The determination of the sampling frequency for the structural health monitoring system of small aircraft digital twins has been demonstrated; that is, a sampling frequency of no less than 1000 Hz is recommended to ensure reliable aircraft damage assessment. The hardware for the MMFS was designed, including an MCU, strain gauge modules, 3D accelerometers, PZT sensors, and thermistors. A prototype was developed. The software has been designed to enable each sensor node to acquire data from the four types of sensors continuously and in real-time. The calibrations and core data processing/communication functionalities of the MMFS have been tested in the laboratory, where loads were applied using a 250 kN Instron hydraulic fatigue testing machine. In addition, we successfully pre-processed the measured raw signals, reducing noise in the data. The analysis of the results indicates that the proposed hardware and data acquisition system can be utilised as the MMFS, demonstrating its potential for effective real-time and continuous data collection in aircraft structural health monitoring.

## Figures and Tables

**Table 1:** Summary of system-level bandwidth, latency, and data-integrity characteristics.

Module	Function	Bandwidth	Latency	Data Integrity/Jitter	Parameter Source
**Analogue Front-End (Strain + filter)**	Signal conditioning and anti-aliasing filter	0.1–81 Hz	<1 ms	High input impedance, shielded wiring	Design parameter
**Analogue Front-End (PZT conditioning)**	Signal conditioning only	--	<1 ms	Shielded wiring	Design parameter
**Analogue Front-End (Accelerometer module)**	Signal conditioning and anti-aliasing filter	1.5 kHz [48]	<1 ms	Noise density = 80 µg/√Hz [48]	Design parameter and datasheet
**ADC + DMA (Double-buffer)**	Analogue-to-digital conversion and buffered data transfer	0–500 Hz	<10 µs	DMA overflow monitored; hardware-triggered sampling ensures continuity	Design parameter and datasheet
**Wireless Communication (BLE/Wi-Fi)**	Data transmission	Up to 500 kbps	10–30 ms	checksum mechanisms; retry protocol	Datasheet
**Storage/Cloud**	Logging and Backup	N/A	<50 ms	File checksum verification	Datasheet

**Table 2:** Corresponding components and parameters of the strain gauge amplifier circuit (Figure 7b).

Component	Value/Type
**C3, C4**	1 µF
**C2**	0.1 µF
**C1**	220 μF/10 V
**R1**	3.48 KΩ
**R2**	1.96 KΩ
**R3**	3 KΩ
**R4, R5, R13**	348 Ω
**R6, R11**	348 KΩ
**R7, R10**	6.98 KΩ
**R8**	9.66 KΩ
**R9**	348 KΩ
**R12**	11 Ω
**RP1 Trimmer Potentiometers**	1 KΩ
**U1B, U1A Dual Rail-to-Rail CMOS Operational Amplifier**	3PEAK TP10-2
**U3 Programmable Precision Shunt Regulator**	TL431
**LED1**	0805LED

## Data Availability

The data will be made available upon reasonable request.
